# Omics analyses of *Rehmannia glutinosa* dedifferentiated and cambial meristematic cells reveal mechanisms of catalpol and indole alkaloid biosynthesis

**DOI:** 10.1186/s12870-023-04478-3

**Published:** 2023-10-05

**Authors:** Pengfei Zhou, Haihua Li, Yujin Lin, Yujun Zhou, Yinzi Chen, Yiheng Li, Xuan Li, Hui Yan, Weiming Lin, Beilu Xu, Huiting Deng, Xiaoqi Qiu

**Affiliations:** 1 School of Basic Medical Science, Guangdong Medical University, Dongguan, 523808 China; 2 School of Medicine and Health, Guangdong Innovative Technical College, Dongguan, 523946 China; 3 School of Pharmacy, Guangdong Medical University, Dongguan, 523808 China

**Keywords:** Cell suspension cultures, Cambial meristematic cells, Catalpol, Iridoids, Metabolome, Transcriptome, Plant-hormone signal transduction, Tryptophan, Phenylalanine

## Abstract

**Background:**

*Rehmannia glutinosa* is a rich source of terpenoids with a high medicinal reputation. The present study compared dedifferentiated cells (DDCs) and cambial meristematic cells (CMCs) cell cultures of *R. glutinosa* for terpenoid (catalpol) and indole alkaloid (IA) biosynthesis. In this regard, we used widely targeted metabolomics and transcriptome sequencing approaches together with the comparison of cell morphology, cell death (%), and catalpol production at different time points.

**Results:**

We were able to identify CMCs based on their morphology and hypersensitivity to zeocin. CMCs showed higher dry weight content and better catalpol production compared to DDCs. The metabolome analysis revealed higher concentrations of IA, terpenoids, and catalpol in CMCs compared to DDCs. The transcriptome sequencing analysis showed that a total of 27,201 genes enriched in 139 pathways were differentially expressed. The higher catalpol concentration in CMCs is related to the expression changes in genes involved in acetyl-CoA and geranyl-PP biosynthesis, which are precursors for monoterpenoid biosynthesis. Moreover, the expressions of the four primary genes involved in monoterpenoid biosynthesis (*NMD*, *CYP76A26*, *UGT6*, and *CYP76F14*), along with a squalene monooxygenase, exhibit a strong association with the distinct catalpol biosynthesis. Contrarily, expression changes in *AADC*, *STR*, and *RBG* genes were consistent with the IA biosynthesis. Finally, we discussed the phytohormone signaling and transcription factors in relation to observed changes in metabolome.

**Conclusions:**

Overall, our study provides novel data for improving the catalpol and IA biosynthesis in *R. glutinosa*.

**Supplementary Information:**

The online version contains supplementary material available at 10.1186/s12870-023-04478-3.

## Background

The Chinese Foxglove (*Rehmannia glutinosa* L.) is an important member of the Scrophulariaceae family and is considered to be one of the 50 fundamental herbs in traditional Chinese medicine [1]. It is widely distributed in central China where it is mainly cultivated (mostly in Henan province) but wild populations are also found up to 1100 m above sea level [2, 3]. The *R. glutinosa* has a range of health benefits and was described in “Shennong’s Herba” [2, 4]. *R. glutinosa* health benefits appear to relate to the presence of glycosides, saccharides (mono, oligo, and polysaccharides), iridoids (and mainly catalpol, dihydrocatalpol, and acetylcatalpol), and monoterpenoids (e.g. rehmanniosides) [2, 5, 6].

Iridoids are a large family of monoterpenoids. They are produced from geraniol, which originates from a common biosynthesis route involving non-mevalonate and mevalonate pathways. Research on *Catharanthus roseus* [4], *Picrorhiza kurroa* [5], and *R. glutinosa* [6] has brought us some missing links in the iridoid biosynthesis (especially the catalpol). Major genes, that have been identified in iridoid (catalpol) biosynthesis in *R. glutinosa,* are included in terpenoid backbone biosynthesis and monoterpenoid biosynthesis pathways [6] as they are a group of monoterpenoids. Catalpol and loganin are representatives of carbocyclic iridoids. Geranyl-PP is the starting point of the monoterpenoid biosynthesis pathway, which is converted to geraniol by the action of geranyl diphosphate diphosphatase. The geraniol is subsequently converted into iridotrial by the action of six genes/enzymes. Which is further converted to loganate (https://www.genome.jp/pathway/map00902; accessed on 05/04/2023) and then to catalpol [7]. Nevertheless, the need to explore the key genes involved in the biosynthesis of these health-beneficial compounds in *R. glutinosa* drives novel research. Additionally, the search for the important transcriptional regulators i.e., transcription factors (TFs) is evident to establish sustainable iridoid production systems. Since plants grow slower and the above-described molecules are produced in lower quantities as compared to total plant biomass, Therefore, multiple strategies are adapted to extract these compounds (especially iridoids in the case of *R. glutinosa*) [8]. These strategies include natural harvest from source plants, semi or complete chemical synthesis from precursors, and tissue culture. Of these strategies, plant cell culture is perhaps the most sustainable system with the advantages of relatively better control of the biosynthesis (conditions and types of cells), environment friendliness, and robustness [9].

At present, the in vitro plant cell and tissue culture of *R. glutinosa* i.e., callus and hairy root culture, are mainly composed of dedifferentiated cells (DDCs). However, studies in multiple plant species have demonstrated that epigenetic changes lead to the loss of DNA methylation of transposable element activators and can pass on to two sexual generations. Similarly, in Arabidopsis cell suspensions, the dedifferentiation and calluses showed hypermethylation of promoters of several genes. Thus, the utility of long-term DDC cultures for the biosynthesis of desired metabolites, such as indole alkaloids (IAs) [10] and iridoids, falls short of being ideal. Additionally, studies have reported slower cell growth, weaker shear resistance, and lower secondary metabolite content in DDCs [11]. Contrarily, the stem cell culture system from root cambial induction (also known as cambial meristematic cells or CMCs) is a relatively better platform for the biosynthesis of plant natural products due to their benefits such as robustness, continuous biosynthesis/supply, easy extraction protocols, environmental friendly, and higher metabolite yields [12]. Overall, we can say that metabolite biosynthesis through CMCs is regarded as a “Good Manufacturing Practice” [9].

Our laboratory has established *R. glutinosa* CMCs obtained from the root cambial induction. These CMCs are kind of primitive, undifferentiated, and have the ability to divide and proliferate indefinitely. Additionally, CMCs can differentiate into diverse types of cells and tissues. Recent studies in *Tripterygium wilfordii*, *Ocimum basilicum*, and *C. roseus* have shown higher metabolite biosynthesis in CMCs as compared to DDCs [10, 13, 14]. Considering these studies, we hypothesize that *R. glutinosa* CMCs would have better metabolite biosynthesis (especially iridoids, e.g., catalpol). To determine the overall differences in metabolite biosynthesis in *R. glutinosa* DDCs and CMCs, we performed a global metabolome analysis. Furthermore, we also performed transcriptome sequencing of the DDCs and CMCs to understand the possible transcriptomic signatures associated with the differential metabolite profiles of both types of cell cultures.

## Results

### Differences in anatomy, cell growth, and catalpol production in *R*. *glutinosa* DDCs and CMCs

**Fig. 1:**
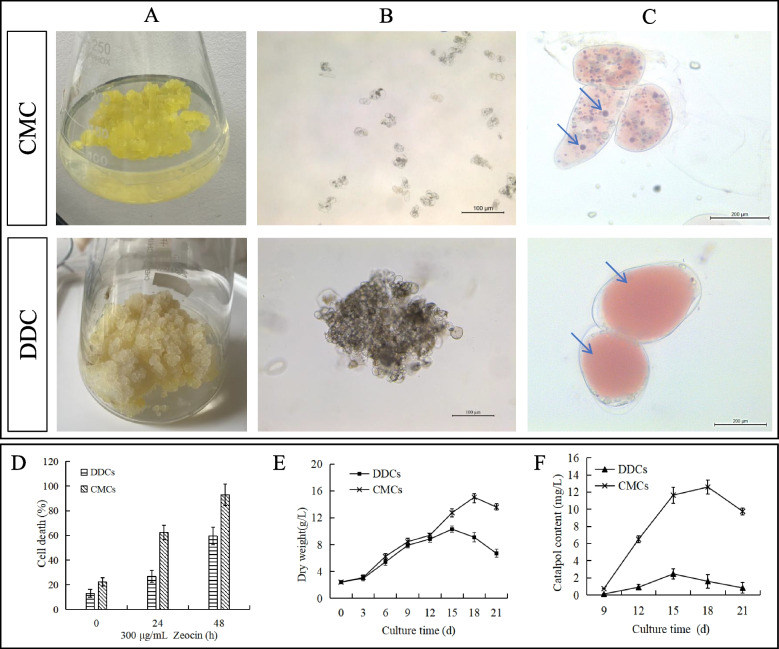
Differences in *R*. *glutinosa* CMCs and DDCs. **A** visual differences in cell cultures, **B** micrographs of CMCs and DDCs under a light microscope, **C**) micrographs of CMCs and DDCs showing vacuoles (blue arrows). The figure panels **B** and **C** are supplemented with a scale bar. **D** Cell death %, E) changes in growth (g/L) over time, and F) catalpol content (mg/L). The values in figure panels D-F are means (*n* = 3) ± SD

The *R. glutinosa* CMCs obtained from roots formed a layer of cells that seemed soft in texture and yellow-green in color. Differently, the DDCs obtained from the roots seemed hard masses and light yellowish in color (Fig. 1A). The microscopic observations showed that the CMC cell culture contained mostly single cells or clusters of a relatively smaller number of cells, whereas, in case of DDCs, the cell culture was seen as dense and large clusters containing a very high number of cells (Fig. 1B). Anatomically, the CMCs contained small and many vacuoles, whereas the DDCs contained one large vacuole which covered most of the area of the cell (Fig. 1C).

The cell death % of CMCs was notably higher as compared to DDCs, which indicates hypersensitivity of CMCs in response to the radiomimetic antibiotic zeocin, which is consistent with the earlier findings [15] (Fig. 1D). Regarding cell growth, CMCs dry weight increased over time, reaching a maximum at 18 h (Fig. 1e). The growth of DDCs instead peaked at 15 h (Fig. 1E). These data indicate that CMCs perform better in terms of dry weight accumulation and can grow for a longer time compared to DDCs. Finally, the catalpol content (mg/L) of DDCs was lower than that measured in CMCs, at all time points (apart from the ‘9 h’ time point, i.e. when catalpol production started; Fig. 1F). Overall, these results show that CMCs outperform DDCs in terms of growth capacity, dry weight, and catalpol biosynthesis.

### Comparative metabolome of *R*. *glutinosa* DDCs and CMCs

**Fig. 2:**
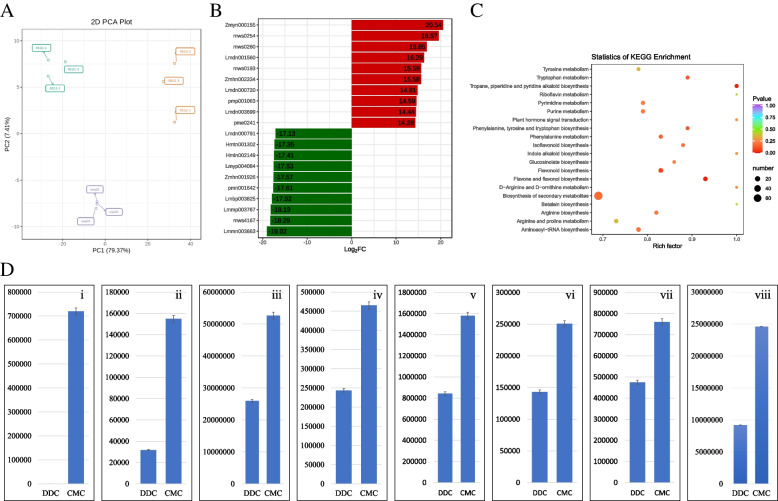
Details of metabolomics features for DDCs (REG-1) and CMCs (REG-2 samples). **A** Principal Component Analysis score chart based on mass spectrum data of DDCs and CMCs, and QC samples (mix). Ordinate: the second principal component, Abscissa: the first principal component. **B** Top 20 FC change metabolites. Ordinate: metabolite, Blackish green color: down-regulated metabolites, Red: up-regulated metabolites. **C** Scatter plot showing top pathways to which differentially expressed genes/transcripts were significantly enriched. The ordinate represents the KEGG pathway. The abscissa represents the Rich factor. **D** Relative content of (i) 6-deoxycatalpol, (ii) dihydrocatalpol, (iii) acetylcatalpol, (iv) catalpol, (v) specioside (6-o-*p*-coumaroylcatalpol), (vi) 10-o-(4-hydroxycinnamoyl)-6'-o-β-D-glucosylcatalpol, and (vii) 6-o-veratroylcatalpol in DDCs and CMCs. The data bars show the relative content on Y-axis. (viii) The relative total content of alkaloids (presented on Y-axis) in CMCs and DDCs. The error bars represent the standard deviation

In the current study, a total of 756 metabolites were identified from two sources: REG-1 (DDCs) of *Rehmannia glutinosa* and REG-2 (CMCs) of *R. glutinosa*. These metabolites belong to 12 major classes, including lipids (123 metabolites), flavonoids (122 metabolites), phenolic acids (106 metabolites), terpenoids (88 metabolites), others (73 metabolites), amino acids and derivatives (69 metabolites), organic acids (61 metabolites), alkaloids (43 metabolites), nucleotides and derivatives (43 metabolites), lignans and coumarins (19 metabolites), tannins (07 metabolites), and quinones (01 metabolite). A detailed list of these metabolites is provided in Supplementary Table 1. The Principal Component Analysis (PCA) score plots of metabolites from DDCs and CMCs were produced to evaluate the differences in the whole metabolome between both types of samples (Fig. 2A). The metabolic profile analysis showed a clear separation of the DDC group from the CMC group, indicating significant changes in the metabolic profiles between the two groups. To further elucidate the metabolic profile differences between the two types of samples, a differential clustering analysis was performed (Supplementary Table 2).

Out of 756 annotated metabolites, 510 metabolites were significantly differentially accumulated, with 349 down-regulated and 161 up-regulated (Supplementary Table 2). The top-20 metabolites with the highest fold changes are presented in Fig. 2B, including 10 up-regulated and 10 down-regulated metabolites. The top-10 down-regulated metabolites are representative of various classes, such as phenolic acids (glucosyloxybenzoic acid, acteoside, 1-o-salicyl-D-glucose, 5-glucosyloxy-2-hydroxybenzoic acid methyl ester), flavonoids (kaempferol-3-o-(2''-o-acetyl)glucuronide, kaempferol-7-o-glucuronide, luteolin-7-o-glucuronide), coumarins (skimmin (7-hydroxycoumarin-7-o-glucoside)), and terpenoids (rehmaglutin C, perillyl alcohol). On the other hand, the top 10 up-regulated metabolites include amino acids/derivatives (N-α-acetyl-l-ornithine, L-histidine, L-arginine, L-homocitrulline), phenolic acids (6-o-fruloyl-D-glucose, benzoic acid), and terpenoids (6-deoxycatalpol, rehmannioside B, 7'-o-sinapoyljasminoside L, 6-o-trans-caffeoyl ajugol).

When all the differentially regulated metabolites (510) were annotated using the Kyoto Encylopedia of Genes and Genomes (KEGG) database, 188 metabolites were successfully matched to KEGG pathways. Based on the KEGG classification, these metabolites were found to be associated with 138 metabolic pathways, 77 pathways for biosynthesis of secondary metabolites, and 27 pathways for biosynthesis of amino acids. Notably, the KEGG enrichment analysis revealed that pathways related to secondary metabolites showed enrichment, as depicted in Fig. 2C.

In the current study, catalpol and rehmannioside are considered the quality control compounds of *R. glutinosa*. During our investigation, we successfully identified several compounds, including rehmanniosides (B, C, and D), catalpol, acetylcatalpol, specioside (6-o-*p*-coumaroylcatalpol), 6-o-veratroylcatalpol, 10-o-(4-hydroxycinnamoyl)-6'-o-β-D-glucosylcatalpol, dihydrocatalpol, and 6-deoxycatalpol. Overall, the accumulation of these compounds (especially catalpol) was higher in CMCs as compared to DDCs (Fig. 2Di). Furthermore, we observed that total IAs content in the CMCs was higher than in the DDCs (Fig. 2Dii).

Among plant hormones, abscisic acid (ABA), salicylic acid (SA), salicylic acid-2-o-glucoside, and (-)-jasmonoyl-L-isoleucine were found to be down-regulated (Supplementary Table 2). In the starch and sucrose metabolism pathway, D-sucrose, uridine 5'-diphospho-D-glucose, and D-glucose 1,6-bisphosphate were up-regulated, while D-glucose was down-regulated. Regarding the phenylalanine, tyrosine, and tryptophan biosynthesis pathway, L-tryptophan, indole, L-phenylalanine, D-fructose-1,6-biphosphate, L-tyrosine, and quinic acid were up-regulated in CMCs. On the other hand, phenylpyruvic acid and 3-hydroxybenzoic acid were down-regulated in the same pathway (Supplementary Table 2).

These results suggest that CMCs exhibit relatively higher contents of catalpol (and related compounds), rehmanniosides, IAs, and sucrose compared to DDCs. The changes in phytohormone concentrations indicate that the signaling in both cell types could be related to the alterations in differential metabolite contents. Consequently, we conducted further investigations to explore the key transcriptomic signatures associated with catalpol and alkaloids in both types of cells.

### Transcriptome analyses of *R*. *glutinosa* CMCs and DDCs

#### Transcriptome sequencing

**Fig. 3:**
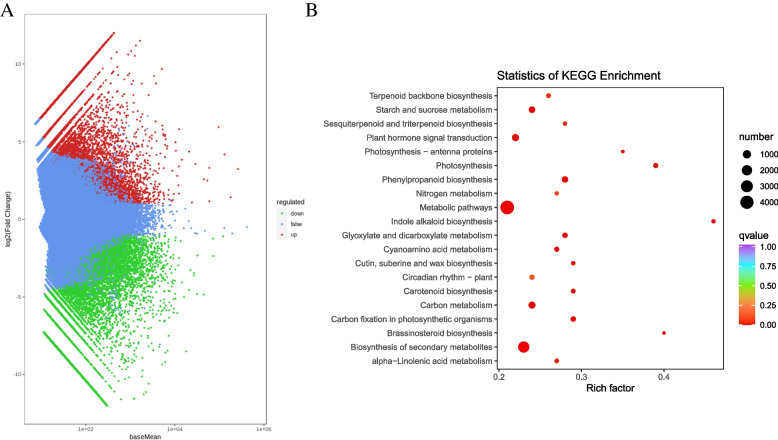
**A** Volcano map showing differentially expressed genes between *R. glutinosa* DDCs and CMCs. **B** Scatter plot showing pathways to which the DETs were significantly enriched in DDC vs. CMC

In this project, a total of six samples (three for each cell type) were subjected to transcriptome sequencing, resulting in a total of 28.26 Gb clean data. Each sample’s clean data reached 6 Gb, with the percentage of Q30 bases at 93% and above. The average sequencing error rate was 2.5 and the guanine-cytosine content was approximately 45%. Out of the 253,440 transcripts, 201,026 were successfully assembled into unigenes, as shown in Fig. 3A, with N50 and N90 values of 1408 and 468, respectively (Supplementary Table 3). All the unigenes obtained from the assembly were subjected to annotation, with the following annotation rates for various databases: KEGG (49.25%), non-redundant (NR, 68.22%), SwissProt (47.92%), Eukaryotic Orthologous Groups of Proteins (KOG, 39.98%), gene ontology (GO, 54.21%), and Pfam (48.61%) (Supplementary Fig. 1).

#### Differential gene expression between DDCs and CMCs

##### Differential gene expression between DDCs and CMCs confirm metabolite results related to terpenoid, and indole alkaloid accumulation

A total of 27,201 genes exhibited differential expression between DDCs and CMCs, with 14,738 genes upregulated and 12,463 genes downregulated in CMCs compared to DDCs (Fig. 3A). Based on the analyses of differential metabolite profiles and KEGG pathway enrichment (Fig. 3B), we specifically focused on changes in the expression of differentially expressed genes/transcripts (DEGs/DETs) involved in the following pathways: glycolysis (73 DETs), terpenoid backbone biosynthesis (73 DETs), monoterpenoid biosynthesis (64 DETs), IA biosynthesis (73 DETs), and tryptophan biosynthesis (73 DETs) pathways (including phenylalanine, tyrosine, and tryptophan biosynthesis (69 DETs)) (Supplementary Table 4).

**Fig. 4:**
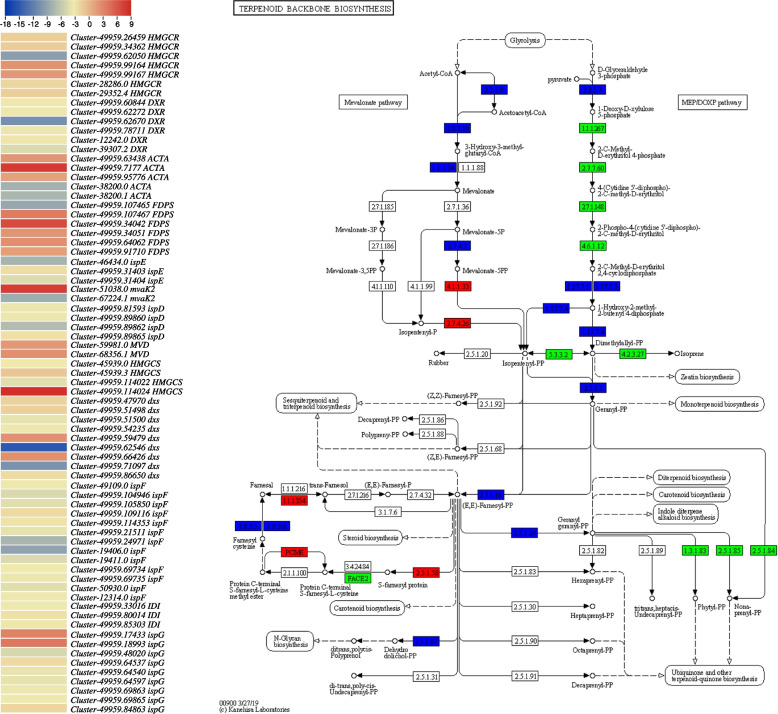
Heatmap of log2FC values of the differentially expressed genes between *R. glutinosa* DDCs and CMCs that were enriched in the terpenoid backbone biosynthesis pathway. The heatmaps were prepared in TBtools [16]. The full names and FPKM values of these genes can be accessed in Supplementary Table 4. The second panel shows the genes that were up (red), down (green), or variably (blue) regulated in the terpenoid backbone biosynthesis pathway. The pathway map was prepared by using KEGG PATHWAY Database [17]

The DEGs/DETs enriched in the glycolysis pathway consisted of 9 genes. Among these genes, the *alcohol dehydrogenase* (*ALD*) *S-(hydroxymethyl) glutathione dehydrogenase/alcohol dehydrogenase (ADH5/ALD)*, *L-lactate dehydrogenase*, *glyceraldehyde 3-phosphate dehydrogenases*, and *pyruvate dehydrogenase E1 component alpha subunits* showed increased expressions in CMCs as compared to DDCs. These changes indicate that the biosynthesis of acetyl-CoA is enhanced in CMCs as compared to DDCs, which is a precursor for monoterpenoid biosynthesis (Fig. 4). The DETs in terpenoid backbone biosynthesis pathway were annotated as 13 genes, with five genes involved in the mevalonate pathway branch and six genes related to the MEP/DOXP pathway branch (non-mevalonate). Interestingly, most of the DETs on the non-mevalonate side of the pathway were down-regulated in CMCs compared to DDCs, except for *(E)-4-hydroxy-3-methylbut-2-enyl-diphosphate synthase* (*Cluster-49959.17433*, and *Cluster-49959.18993*) and *1-deoxy-D-xylulose-5-phosphate synthase* (*Cluster-49959.59479*, and *Cluster-49959.66426*). On the mevalonate side of the pathway, of the five *acetyl-CoA C-acetyltransferase* transcripts, two (*Cluster-49959.63438* and *Cluster-49959.95776)* were highly up-regulated in CMCs, while another (*Cluster-49959.7177*) was exclusively expressed in CMCs. Indicating, the conversion of acetyl-CoA to acetoacetyl-CoA. The upregulation of two *diphosphomevalonate decarboxylase* transcripts (*Cluster-59981.0*, and *Cluster-68356.1*) in CMCs compared to DDCs is an intriguing finding that suggests an increased biosynthesis of mevalonate-5PP (Fig. 4; Supplementary Table 4). This upregulation indicates enhanced production of acetoacetyl-CoA through the conversion of acetyl-CoA. Moreover, the upregulation of five out of the six transcripts of *farnesyl diphosphate synthase* is important since this enzyme converts the precursors from both branches of the pathway to geranyl-PP. Geranyl-PP serves as a crucial starting point for monoterpenoid biosynthesis (Fig. 4).

**Fig. 5:**
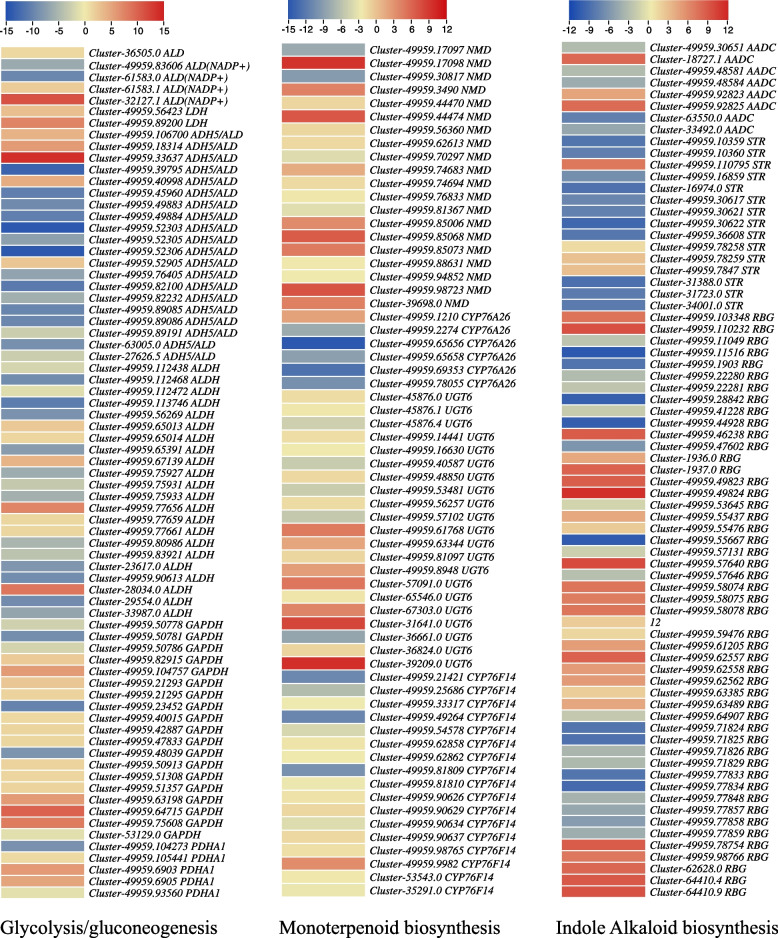
Heatmaps of log2FC values of the differentially expressed genes between *R. glutinosa* DDCs and CMCs that were enriched in the glycolysis/gluconeogenesis, monoterpenoid biosynthesis, IAs biosynthesis pathways. The full names and FPKM values of these genes can be accessed in Supplementary Table 4. The heatmaps were prepared in TBtools [16]

Moving downstream, the DETs enriched in monoterpenoid biosynthesis were annotated as four major genes i.e., *(* +*)-neomenthol dehydrogenase* (*NMD*), *cytochrome P450 family 76 subfamily A* (*CYP76A26*), *7-deoxyloganetin glucosyltransferase* (*UGT6*, also known as *Iridoid glucosyltransferase*), and *(E)-8-carboxylinalool synthase* (*CYP76F14*). Among these genes, the upregulation of *CYP76A26* (*Cluster-49959.1210*) and *UGT6* (*Cluster-49959.61768*, *Cluster-57091.0*, *Cluster-31641.0*, and *Cluster-39209.0*) is particularly relevant to the observed changes in loganin (Fig. 5; Supplementary Table 4). This relevance was further confirmed by the observation that loganin, loganin acid, and 7-deoxyloganic acid showed a correlation with the genes mentioned above (Supplementary Fig. 2A). These findings indicate the potential involvement of these key genes in the biosynthesis of loganin and related monoterpenoids, contributing to the differences observed in metabolite profiles between CMCs and DDCs in *R. glutinosa*.

Indeed, the conversion of aucubin to catalpol involves the action of a squalene monooxygenase (SQM) enzyme. Interestingly, our study revealed the upregulation of a *SQM* gene (*Cluster-65738.5*) in CMCs compared to DDCs (Supplementary Table 4). These observed expression changes suggest that the higher production of catalpol and iridoids in CMCs can be attributed to the upregulation of multiple genes enriched in glycolysis, terpenoid backbone biosynthesis, and monoterpenoid biosynthesis.

The DETs enriched in IAs biosynthesis were annotated as three major genes: *aromatic-L-amino-acid/L-tryptophan decarboxylase* (*AADC*), *strictosidine synthase* (*STR*), and *raucaffricine beta-D-glucosidase* / *vomilenine glucosyltransferase* (*RBG*). Three of eight AADCs, four of 15 STRs, and 26 of 50 RBGs were up-regulated in CMCs as compared to DDCs. These expression changes are consistent with the observed differences in the accumulation of alkaloids, where 17 out of 30 alkaloids were up-regulated in CMCs as compared to DDCs (Fig. 5; Supplementary Table 4). These findings suggest that these key genes involved in IA biosynthesis can be manipulated to enhance alkaloid production in both types of cells, providing potential targets for improving alkaloid biosynthesis in *R. glutinosa.*

##### Transcriptome sequencing results confirm tryptophan and L-phenylalanine accumulation patterns in DDCs and CMCs

**Fig. 6:**
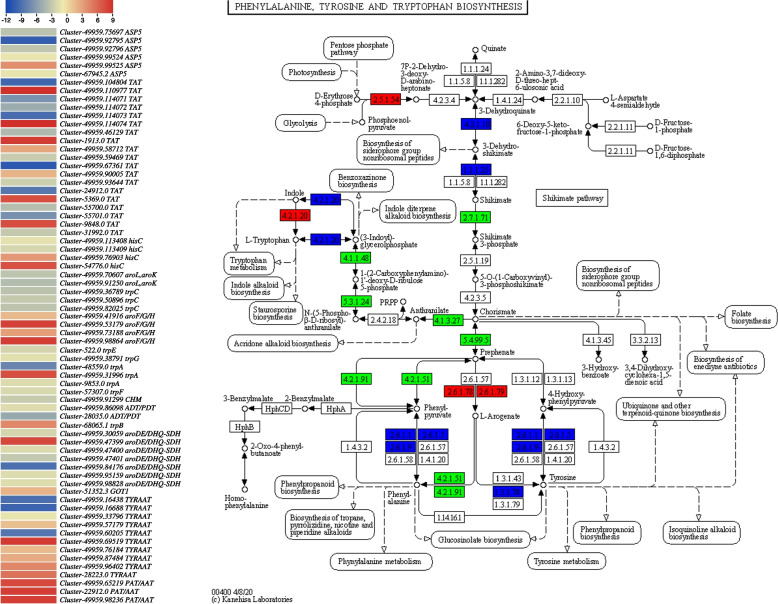
Heatmap of log2FC values of the differentially expressed genes between *R. glutinosa* DDCs and CMCs that were enriched in the phenylalanine, tyrosine, and tryptophan biosynthesis pathway. The heatmaps were prepared in TBtools [16]. The full names and FPKM values of these genes can be accessed in Supplementary Table 4. The second panel shows the genes that were up (red), down (green), or variably (blue) regulated in the phenylalanine, tyrosine, and tryptophan biosynthesis pathway. The pathway map was prepared by using KEGG PATHWAY Database [17]

Since the metabolome analysis revealed an increased accumulation of both L-phenylalanine and L-tryptophan, we further explored the expression trends of genes enriched in the associated pathways. A total of 69 and 73 DETs were enriched in phenylalanine, tyrosine (Fig. 6), and tryptophan biosynthesis and tryptophan metabolism pathways, respectively. The AADCs were commonly found between IA biosynthesis and tryptophan metabolism pathways. Moreover, the major genes in tryptophan metabolism were *aldehyde dehydrogenase* (*ALDH*) and *amidase*. Two *amidases* (*Cluster-49959.62218* and *Cluster-49959.70278*) were highly expressed in both cell types and showed increased expressions in CMCs. Similarly, of all ALDHs, *Cluster-28034.0* and *Cluster-49959.77656* were exclusively expressed in CMCs. Additionally, *Cluster-49959.65014*, *Cluster-49959.67139*, and *Cluster-49959.77659* showed the highest expressions among all the ALDHs in CMCs. The expression changes in these genes correspond to L-tryptophan accumulation pattern in CMCs as compared to DDCs. The fact that we observed a correlation between these DETs and tryptamine and L-tryptophan, further strengthens our statements (Supplementary Fig. 2B). In the phenylalanine, tyrosine, and tryptophan metabolism, the upregulation and/or exclusive expression of *3-deoxy-7-phosphoheptulonate synthase*, *tryptophan synthase alpha chain*, and *3-dehydroquinate dehydratase/shikimate dehydrogenase* correspond to the changes in L-phenylalanine (Fig. 6; Supplementary Table 4). Here the CMC and DDC-specific genes in this pathway can be useful for L-phenylalanine biosynthesis in both types of cells.

##### Expression changes in signaling-related pathways

The differential regulation of a considerable number of transcripts (428 annotated as 32 genes) enriched in starch and sucrose metabolism is a significant finding. The observed decrease in glucose and increase in sucrose accumulation in CMCs compared to DDCs is consistent with the upregulation of certain key genes. The upregulation of *glucose-1-phosphate adenylyltransferase large subunit 1 (A)*, *lysosomal beta glucosidase-like (A)*, nineteen transcripts for *sucrose synthase*, *trehalose-phosphate phosphatase A-like (A)* is consistent with observed patterns of sucrose accumulation in CMCs. These genes are involved in sucrose synthesis and are likely contributing to the higher sucrose content in CMCs. On the other hand, the downregulation of a large number of genes associated with glucose breakdown, such as hexokinases and glucose-6-phosphate isomerases, corresponds to the lower glucose content in CMCs. Since sucrose can act as a signal in plant metabolism, these observed changes in gene expression are important and likely have functional implications in the regulatory network of *R. glutinosa* CMCs (Supplementary Table 4).

**Fig. 7:**
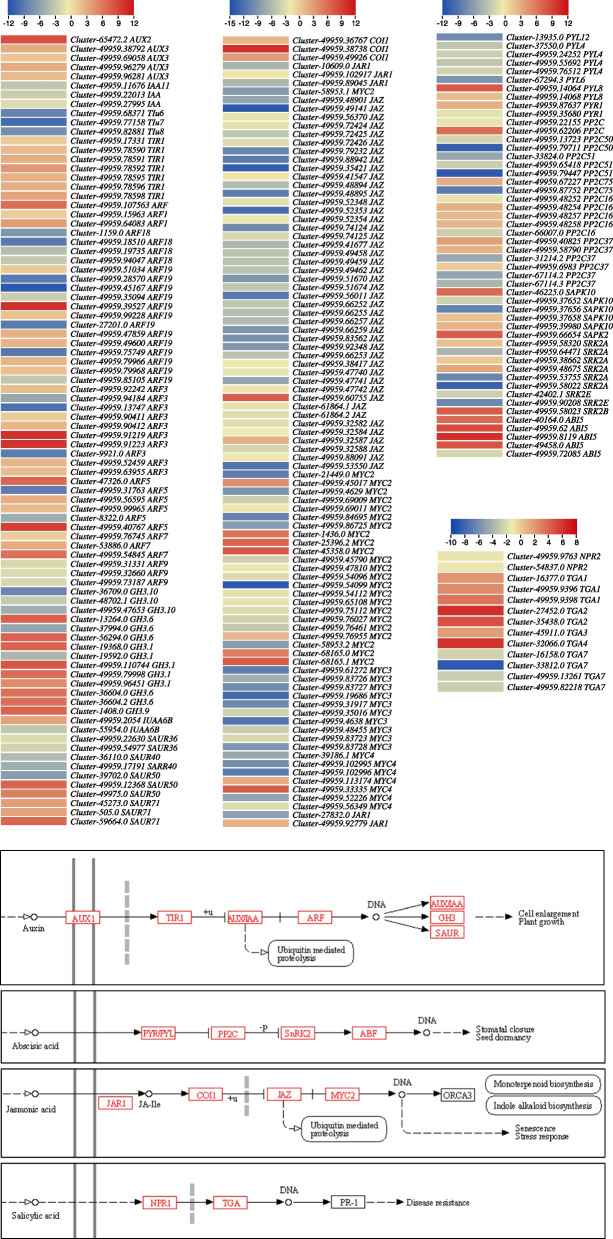
Heatmaps of log2FC values of the differentially expressed genes between *R. glutinosa* DDCs and CMCs that were enriched in the plant-hormone signal transduction pathway. The pathway panels show the genes (in red) which were differentially expressed. The heatmaps were prepared in TBtools [16]. The full names and FPKM values of these genes can be accessed in Supplementary Table 4

In the case of plant-hormone signal transduction pathway, 606 transcripts were annotated as 40 major genes/TFs (Fig. 7). Interestingly, except for six KO terms/genes, all were differentially expressed in the two cell types, indicating a significant role of phytohormone signaling in the observed metabolic changes. As we noted in metabolome results that the metabolites related to three phytohormones (indole-3-acetic acid (IAA), ABA, and SA) were differentially accumulated between CMCs and DDCs. We found that 89 transcripts associated with six auxin signaling-related genes were differentially expressed. Clearly, the *auxin transporter-like protein 2/3* transcripts, *transport inhibitor response 1* (*TIR1*) proteins, auxin response factors (*ARF1, ARF19-like, ARF3, ARF5*, and *ARF7*), *indole-3-acetic acid-amino synthetase* (*GH3.1* and *GH3.6*), and *auxin-responsive protein* (*SAUR50-like* and *SAUR71-like*) transcripts were up-regulated in CMCs. Whereas *auxin-induced protein 22D*, *ARF18*, *ARF19, ARF3, ARF9, GH3.10, SAUR36,* and *SAUR40* transcripts were mostly DDC-specific or down-regulated in CMCs. These expressions indicate auxin-driven signaling in CMCs and DDCs (Fig. 7; Supplementary Table 4).

As for ABA, 57 transcripts belonging to four ABA-signaling genes were differentially expressed. The ABA-receptor (PYL) *PYL12, PYL4, PYL6,* and *PYR1-like* were down-regulated in CMCs. The *PYR1* and *PYL8* were up-regulated in CMCs. *Protein phosphatase 2C* (*PP2C*) transcripts showed variable expressions i.e., the ones expressed in one type of cells were either exclusive or showed minor expressions in other cells. Serine/threonine-protein kinase *SAPK10, SAPK2A, SRK2A, SRK2A-like,* and *SRK2E* showed variable expressions. However, *SAPK2* and *SRK2B-like* were exclusive to CMCs. The *ABA-INSENSITIVE 5-like protein 7* was up-regulated in CMCs (Fig. 7; Supplementary Table 4).

Regarding jasmonic acid (JA) metabolism, we observed a reduced accumulation of Jasmonoyl-L-Isoleucine (JA-Ile) in CMCs compared to DDCs. This observation is consistent with the downregulation of several key genes involved in the JA pathway in CMCs. The *JA-amino synthetase* (*JAR1*), *TIFY 10a-like* (*JAZ*), TF *MYC2*, *MYC2-like, MYC3-like*, and *MYC4-like* were down-regulated in CMCs as compared to DDCs. Conversely, *coronatine-insensitive protein 1-like* (*COI1-like*) transcripts were up-regulated in CMCs as compared to DDCs (Fig. 7; Supplementary Table 4).

Finally, related to SA signaling, we observed upregulation of *TGA1, TGA2, TGA3-like*, and *TGA4-like* in CMCs as compared to DDCs. Whereas, the *TGA7* and *NPR2* were down-regulated in CMCs as compared to DDCs (Fig. 7; Supplementary Table 4).

##### Differentially expressed transcription factors and transcriptional regulators

The comparative analysis showed the differential expression of 1404 TFs or transcription regulators (TRs) classified into 83 families. Out of these, 627 TFs/TRs belonging to 72 families were up-regulated, while 778 TFs/TRs from 70 families were down-regulated in CMCs relative to DDCs. The highest number of transcripts were classified as *AP2/ERF-ERF* family followed by *WRKY*, *bHLH*, *C3H*, *GRAS*, *NAC*, *MYB-related*, *bZIP*, and *AUX/IAA*. Of the TFs/TRs up-regulated in CMCs, 252 were exclusively expressed in CMCs. On the contrary, 221 TFs/TRs were exclusively expressed in DDCs. This indicates that certain TFs/TRs are specific to each cell type. The top-10 highly expressed TFs in CMCs were members of *C3H*, *GARP-G2-like*, *HB-HD-ZIP*, *HB-KNOX*, *HMG*, *LOB*, *TCP*, and *Trihelix* TF families. Whereas the top-10 highly expressed TFs in DDCs belonged to *AP2/ERF-ERF*, *bHLH*, *GRAS*, *SET*, and *Tify* TF families (Supplementary Table 5). The higher up/down-regulation of TFs/TRs between CMCs and DDCs indicates significant transcriptional differences between the two cell types.

### qRT-PCR analyses of selected *R*. *glutinosa* genes

**Fig. 8:**
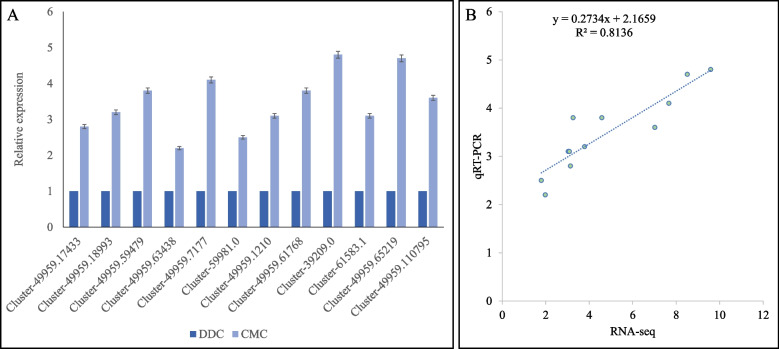
Quantitative real-time PCR analysis of *R. glutinosa* genes in DDCs and CMCs. A) Relative gene expression and B) correlation between FPKM values and relative expression of the selected genes

To validate the RNA sequencing-based expression profiles, we selected 12 genes based on their interesting comparative expression profiles and enrichment in pathways like IA biosynthesis, terpenoid backbone biosynthesis, monoterpenoid biosynthesis, glycolysis/gluconeogenesis, and phenylalanine, tyrosine and tryptophan biosynthesis. The relative expression of these genes was consistent with their FPKM values (Fig. 8A) as evident from higher correction (*R*^2^ > 0.81) between the two types of expression profiles (Fig. 8B). These expression changes also confirm their presented roles in the above-mentioned pathways.

## Discussion

The industrial-scale production of specific metabolites using tissue culture (in vitro cell suspension cultures) techniques has gained significant recognition. In this context, the DDCs and CMCs are generally used for such purposes. It is possible to morphologically distinguish CMCs and DDCs. Our findings regarding the morphological differences are consistent with earlier reports, indicating that CMCs contained smaller multiple vacuoles, which is a characteristic feature of CMCs, in contrast to DDCs, which typically have one large vacuole [15, 18, 19]. Additionally, CMCs were observed either as individual cells or clusters with a relatively smaller number of cells (Fig. 1B). Furthermore, the observation of an increased cell death rate with an increase in exposure time to zeocin supports the notion that the cells under study are CMCs (Fig. 1D). This conclusion is based on the results of previous studies, which have demonstrated that plant stem cells, such as CMCs, are highly sensitive to radiopharmaceuticals like zeocin [13, 20]. The major advantage of CMCs lies in their stem-cell-like properties, which contribute to better growth (Fig. 1E), and sustained homogeneity even after numerous generations [21]. Therefore, CMCs can be conveniently used for in vitro production of specific metabolites. Catalpol is one of the criteria for evaluating the medicinal effects of *R. glutinosa* [22]. In the current study, a higher content of catalpol and its derivative metabolites was recorded in CMCs (Figs. 1F; 2D; Supplementary Table 2). The concentration of catalpol can vary in tissues and organs of *R. glutinosa* as well as at different developmental stages. Additionally, some of the related metabolites may accumulate only in specific tissues or organs [23, 24]. Therefore, based on many features analyzed in this study, it can be stated that CMCs of *R. glutinosa* are a better platform for the production of catalpol and related metabolites compared to DDCs.

**Fig. 9:**
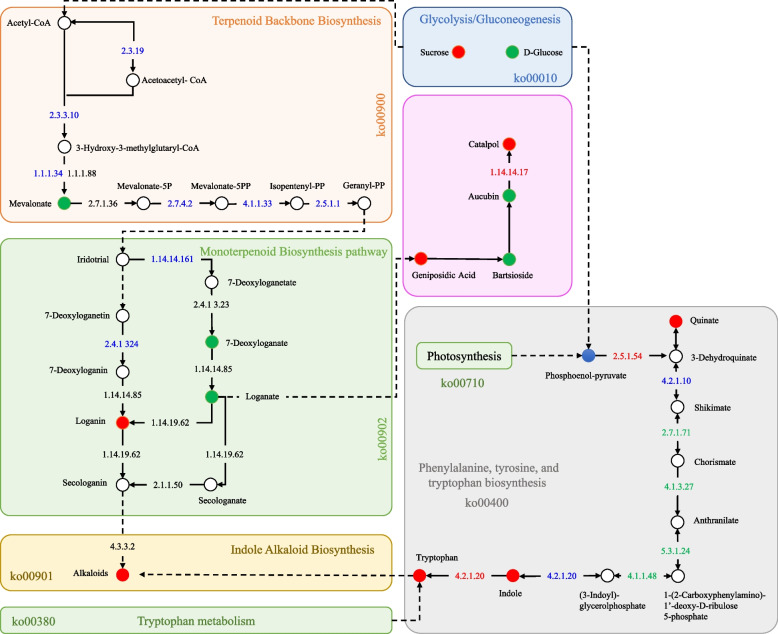
Proposed model of the catalpol biosynthesis and related pathways in *R. glutinosa* DDCs and CMCs. The red, green, and blue colors represent upregulation, downregulation, and up/down-regulation of genes/metabolites, respectively. The pathways are drawn according to KEGG pathway database [31]. The gene names are represented with the E.C. as annotated in KEGG database (See the genes’ details in Supplementary Table 4). The dotted lines show multiple reaction steps, which were not differentially regulated according to the transcriptome comparison results

Since catalpol is produced as a product of terpenoid biosynthesis as well as the upstream pathways [6], the enrichment of DETs and differentially regulated metabolites in terpenoid biosynthesis-related pathways is relevant (Figs. 1C; 3B). The finding that acetyl-CoA-related genes were highly expressed (acetyl-CoA being a precursor for monoterpenoid biosynthesis) might indicate their role in increasing the biosynthesis of catalpol and related metabolites. This is further supported by the reports on the roles of *ALD* [25], *L-lactate dehydrogenase*, *pyruvate dehydrogenase E1 component alpha subunits 1*, and *glyceraldehyde 3-phosphate dehydrogenases* [26]. Both the mevalonate and MEP/DOXP pathways take part in terpenoid (and specifically catalpol) biosynthesis [27]. The observation that a significant portion of genes within the mevalonate pathways were up-regulated indicates their potential involvement in downstream terpenoid (catalpol) biosynthesis (Supplementary Table 4; Fig. 7). In addition to these genes, the higher expressions of *acetyl-CoA C-acetyltransferase*, *diphosphomevalonate decarboxylase*, and *farnesyl diphosphate synthase* further support our hypothesis because of their function in step-wise conversion of Acetyl-CoA to GPP [28, 29]. Indeed, the genes involved in mevalonate and MEP/DOXP pathways as well as those involved in the step-wise conversion of Acetyl-CoA to GPP play critical roles in controlling the biosynthesis of metabolites present upstream of the monoterpenoid biosynthesis pathway [30]. Particularly, *NMDs*, *CYP76A26*, *UGT6*, and *SQM* genes could play major roles in increasing catalpol biosynthesis (Supplementary Table 4; Fig. 9), which further supports our hypothesis. This agrees with a previous study on catalpol biosynthesis which highlighted the relevance of these genes in catalpol biosynthesis in *Centranthera grandiflora* [7] and *R. glutinosa* roots [6]. Thus, we propose that the genes involved in monoterpenoid biosynthesis pathway and its upstream pathway(s) are crucial in controlling catalpol biosynthesis.

In addition to catalpol, improving IA biosynthesis has commercial relevance. CMCs have been proven as a relatively better source of these metabolites e.g., in *C. roseus* [21, 32]. Our findings, overall showing a higher IA biosynthesis in CMCs compared to DDCs, are consistent with those reported in a previous study on *C. roseus* [32]. The differential expression of three genes i.e., *AADC*, *STR*, and *RBG* offers a set of genes that can be manipulated for improvement of IA biosynthesis from CMCs. This is consistent with the functions of *STR* and *AADC* enzymes reported in *C. roseus,* where their overexpression resulted in a greater activity in the IA biosynthetic pathway [33].

Overall, the integrated analysis of metabolome and transcriptome data strongly suggests that CMCs are a more favorable option than DDCs for achieving higher catalpol and IA biosynthesis. The genes associated with monoterpenoid biosynthesis and the upstream pathways, particularly those involved in the mevalonate side of the pathway, appear to be crucial in influencing the differential catalpol and terpenoid biosynthesis (Fig. 9). Moreover, the genes *AADC, STR*, and *RBG* are found to play essential roles in the distinct biosynthesis of IAs in CMCs and DDCs.

The major pathways involved in iridoid biosynthesis, particularly catalpol in this study, are the monoterpenoid biosynthesis and terpenoid backbone biosynthesis pathways. Therefore, understanding the transcriptomic signatures that may influence or regulate the differential biosynthesis of catalpol and alkaloids is of great importance. It has been observed that increased concentration of auxin in CMCs results in the binding of *AUX/IAA* to *TIR1,* leading to the degradation of AUX/IAA proteins [34]. Furthermore, the ARFs were found to be up-regulated in CMCs that activated the transcription of downstream genes i.e., *GH3.1* and *GH3.6* and *SAUR50* and *SAUR71-like* [35]. These changes indicate that CMCs sensed higher auxin concentrations that possibly led to downstream responses. As auxin biosynthesis occurs downstream of the tryptophan pathway, the auxin signaling correlated with the increased tryptophan content in CMCs [36]. How auxin (particularly IAA) signaling regulates terpenoid biosynthesis is not well established yet, but studies have shown simultaneous upregulation of auxin signaling-related genes and *SQM* gene in tomato under abiotic stress, implying a relation between both [37]. Another link could be the common acetyl-CoA molecule present upstream of the auxin biosynthesis, tryptophan biosynthesis, and monoterpenoid biosynthesis pathways (see biosynthesis of plant hormone pathway in KEGG database; https://www.genome.jp/pathway/map01070; accessed on 05/04/2023). Since we found that ABA content diminished in CMCs (Supplementary Table 2), the downregulation of several PYLs, including *PYL12-like*, *PYL4, PYL4-like, PYR1-like*, and *PYL6* was expected [38]. Additionally, the upregulation of *PP2C16-like, PP2C75*, and *PP2C37* suggests their interaction with SnRKs. This was evident from the downregulation of several SnRKs (*SAPK10*, *SRK2A, SRK2A-like*, and *SRK2E*). PP2Cs are negative regulators of SnRKs, therefore, this interaction possibly led to limited or no downstream signaling [39]. However, the contrasting expressions of transcripts with similar annotations indicate that ABA is not completely absent in CMCs and there is a signal transmission. This is evident from the expressions of ABI5 transcripts [38]. The reduced content of JA-Ile in CMCs (Supplementary Table 1) suggests a lower level of JA-Ile biosynthesis in these cells. This observation aligns with the reduced expressions of *JAR1*, an enzyme involved in JA-Ile biosynthesis, in CMCs [40]. The downregulation of *JAR1* likely contributes to the decreased JA-Ile levels observed in CMCs. Furthermore, the upregulation of *COI1-like* genes and the downregulation of *JAZ* genes in CMCs indicate that *COI1*, an essential component of the JA receptor complex, leads to the degradation of JAZ proteins [41]. The degradation of JAZ proteins, which act as repressors of JA signaling, allows the activation of downstream JA-responsive genes. Moreover, the downregulation of *MYC2-like* transcripts in CMCs is noteworthy. *MYC2* is a TF that functions as a negative regulator of JA-mediated responses. Its reduced expression in CMCs implies that *MYC2* is not acting as a JA response terminator in these cells. This observation may have implications for the biosynthesis of monoterpenoids and/or IAs, as *MYC2* can influence the expression of genes involved in these biosynthetic pathways [42]. Much like our findings, previous research in *C. roseus* has highlighted the role of JA signaling in the bio-synthesis of terpenoids and alkaloids [43]. Taken together, the transcriptome analysis reveals simultaneous shifts in expression within terpenoid, alkaloid, phenylalanine, tryptophan, and catalpol biosynthesis pathways, as well as in IAA, ABA, and JA signaling. Future investigations are needed to reveal the influence of these hormones on the synthesis of the aforementioned metabolites.

The possible interaction of TFs with the biosynthesis of alkaloids has been reported in many studies, e.g. the *bHLH*, *AP2/ERF,* and *MYBs* in *C. roseus* [40]*, M. truncatula,* and *Artemisia annua* [41]*.* The differential expression of a large number of TFs in *R. glutinosa* indicates that both the terpenoid and alkaloid biosynthesis is under the transcriptional regulation of many TFs. Similar to our results, an earlier report presented that 59 TF families were differentially expressed in positive/negative relation to catalpol accumulation [6]. It is interesting to see that *AP2/ERF*, *WRKY*, *bHLH*, *C3H*, *GRAS*, *NAC*, *MYB-related*, *bZIP*, and *AUX/IAA* were the most differentially expressed TFs as similar observations have been reported in *C. grandiflora* Benth regarding catalpol biosynthesis [7]. *AP2/ERF* TFs can bind to the promoters of terpenoid IA biosynthesis genes [44]. At the same time, they are JA-inducible, thus establishing a link between phytohormone signaling and alkaloid biosynthesis. Similarly, based on earlier studies showing that WRKYs can positively and negatively regulate discrete classes of metabolites [45], we can expect that a large number of WRKYs are regulating the terpenoid and alkaloid biosynthesis in *R. glutinosa* CMCs and DDCs. The exclusive expression of TFs in both types of cells gives us novel TF candidates to characterize and understand how they regulate the terpenoid and alkaloid biosynthesis in each cell type. Overall, we conclude that the accumulation of terpenoids and alkaloids in *R. glutinosa* involves large number of TFs belonging to diverse families. 

## Conclusions

The current study employed a comprehensive metabolomics and transcriptomics approach to gain insights into the molecular mechanisms involved in terpenoid biosynthesis, with a particular focus on catalpol, in two distinct *R. glutinosa* cell types (CMCs and DDCs). The results showed that CMCs exhibited higher levels of terpenoids, especially catalpol, and related metabolites compared to DDCs. By utilizing the Illumina HiSeq sequencing platform, the transcriptomes of both cell types were sequenced, enabling us to analyze the gene expression patterns and molecular processes underlying the observed metabolic differences. The study revealed significant metabolic changes in both CMCs and DDCs, particularly involving the terpenoid backbone biosynthesis, monoterpenoid biosynthesis, IA biosynthesis, as well as the biosynthesis of tryptophan and L-phenylalanine. These pathways are known to be closely associated with the production of terpenoids and other secondary metabolites in plants. The differential expression of genes/transcripts involved in these biosynthetic pathways could explain the varying terpenoid content between the two cell types. Furthermore, the study suggested that the signaling of important phytohormones, such as IAA, ABA, and JA, might play a role in regulating the biosynthesis of these metabolites in both CMCs and DDCs. The interactions between these phytohormones and the expression of specific genes could influence the production of terpenoids and other secondary metabolites in the cells.

## Methods

### Plant material

Induction of DDCs: Fresh leaves of 1–3 months-old *R. glutinosa* plants were harvested in Jiaozuo, Henan Province, China. No permission is required to work on this species. Voucher specimens are available in the genebank herbarium of Guangdong Medical University, China under the number: GDD230XT99. Official identification of the plant material was conducted by Prof Pengfei Zhou.

Leaf samples were rinsed with tap water for 4 h. After the surface water was absorbed, explant was sterilized with 75% alcohol for 30 s and rinsed with sterile distilled water (5 times). Then, 0.05% (w/v) mercuric chloride solution was applied for 5 min, followed by rinsing with sterile distilled water. This step was repeated five times. Finally, we soaked the explants in 150 mg/l citric acid solution.

The sterilized explants were placed in a glass dish with filter paper, cut into small pieces of 0.5 square centimeters, and affixed to a conical flask containing 50 ml of solid medium for culture. The medium was MS medium containing 30 g/L sucrose, 2 mg/L naphthalene acetic acid, α-naphthalene acetic acid (NAA), 2 mg/L 6-Benzylaminopurine (6-BA), 1 mg/L 2,4-dichlorophenoxyacetic acid 2, 4-Dichlorophenoxyacetic acid. The pH of the solution was adjusted to 6.0. A total of 3 explants were placed in each bottle and transferred to a biochemical incubator for 24 h dark culture. After about 7 to 12 days, it could be observed that small cell clusters grow around the incision of the leaf. After 14 days, they were transferred to the subculture medium for culture. The subculture conditions were 24 h dark culture, temperature 25 °C, and subculture every 12 days. The sample used in this study was named REG-1 (DDCs) of *R. glutinosa* (Fig. 9).

Induction of root CMCs: The fresh annual rhizomes of *R. glutinosa* (Gaert.) Libosch. exFisch. et Mey. were harvested from one-year-old plants growing in Jiaozuo, Henan Province, China. The surface soil of *R. glutinosa* was washed and then rinsed with tap water for 3 h. The cleaned explants were placed on a clean bench, and filter paper was used to absorb the surface water, followed by sterilization with 3% hydrogen peroxide (2 min), rinsing with sterile distilled water (3 times), sterilization with 75% ethanol (2 min), and 3 times rinsing using sterile distilled water. Then, the explants were sterilized for 15 min with mercuric chloride solution (0.1%, w/v), followed by rinsing with sterile distilled water (5 times), and finally the sterilized explants were soaked in 150 mg/l citric acid solution to prevent browning.

**Fig. 10:**
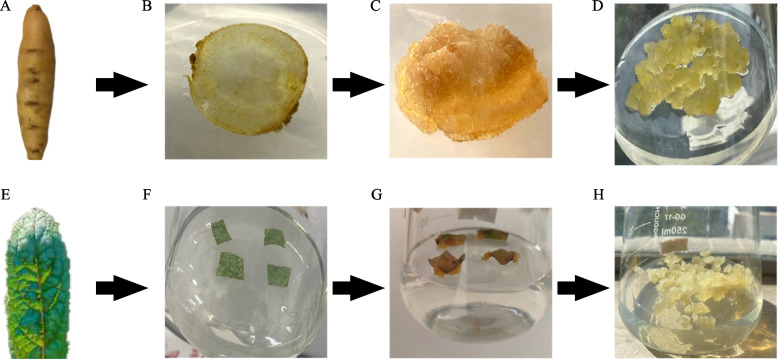
The samples of the *R. glutinosa* CMCs and DDCs were used for sample extraction for metabolome analysis and RNA extraction for transcriptome sequencing and quantitative real-time PCR analyses. **A** one-year-old *R. glutinosa* root, **B** root explant, **C**) CMCs proliferated from the cambium at 12 days culture, **D**) 12-day-old CMCs cultured on solid medium, **E**) three-months-old *R. glutinosa* leaf, **F**) leaf explant, **G**) DDCs proliferated from the leaf at 14 days culture, and **H**) twelve-day-old DDCs cultured on solid medium

The sterilized explants were placed in a glass dish with filter paper, cut into discs with a thickness of 0.5–1.5 mm, and affixed to a conical flask containing 50 ml of solid medium (MS medium) for culture. It contained 30 g/L sucrose, 2 mg/L NAA, 2 mg/L 6-Benzylaminopurine (6-BA), and a pH of 6.0. A total of three explants were placed in each bottle and transferred to a biochemical incubator for 24 h in dark at a temperature of 25 °C. After about 7 to 12 days, a pale-yellow cell mass could be observed around the root cambium of *R. glutinosa*. After 14 days, the newly grown cell mass was transferred to the subculture medium (24 h in dark, 25 ℃ temperature, subculturing every 12^th^ day, the subculture medium was the same as above). The sample used in this study was named REG-2 (CMCs) of *R. glutinosa* (Fig. 10)*.*

To obtain and identify cell suspensions of CMCs and DDCs, 0.1 g of cells were taken, added 1 mL 0.9% normal saline, and pipetted evenly. After that, we took three drops of cell suspension and placed them on a glass slide, and observed under a microscope (Leica DM750). For visualizing differences in anatomy e.g., vacuoles, we took 0.1 g of each cell type, added 1 mL of Ringer solution [46], pipetted the cells evenly, took a drop of cell suspension and transferred to a glass slide, added two drops of 0.25% (w/v) neutral red-ringer solution, and stained the cells for eight minutes. After eight minutes, the dye was blotted with filter paper and the cells were washed twice with Ringer solution followed by adding a drop of Ringer solution on the cells, covered with a cover slide, and observed under a microscope (Leica DM750). For cell death determination, we used radiomimetic drug zeocin as reported earlier [47]. The changes in growth of the CMCs and DDCs were recorded over time (0, 3, 6, 9, 12, 15, 18, and 21 h) and expressed as dry weight (g/L) as reported earlier [13]. Briefly, a 100 mL MS medium containing 2 mg/L NAA, 2 mg/L 6-BA, and 30 g/L sucrose (USA, Caisson) was added in a 250 mL conical flask. The medium was sterilized with high-pressure steam for 20 min, cooled at room temperature, and inoculated with CMCs and DDCs (60 g/L each). The flasks were oscillated at 120 r/min and kept in dark at 25 ℃, while the samples were collected at the time points mentioned above. 

The catalpol content was determined after 9, 12, 15, 18, and 21 h of cell culture growth as reported earlier [22]. 

### Metabolome analyses

#### Sample preparation and extraction

A vacuum freeze-dryer (Scientz-100F) was used to freeze-dry biological samples. A mixer mill with a zirconia bead (MM 400, Retsch; 5 min at 30 Hz)) was employed to crush the freeze-dried sample. A methanol solution (1.2 ml of 70% methanol) was used as a solvent for the lyophilized powder (100 mg), followed by vertexing for 30 s every 30 min. This step was repeated six times. The sample was preserved overnight at 4 °C. Later on, the sample was centrifuged at 12,000 rpm for 10 min. Sample filtration was performed before UPLC-MS/MS analysis (SCAA-104, 0.22 μm pore size; ANPEL, Shanghai, China, http://www.anpel.com.cn/).

#### UPLC conditions

The sample extracts were analyzed using a UPLC-ESI–MS/MS system (UPLC, SHIMADZU Nexera X2, www.shimadzu.com.cn/; MS, Applied Biosystems 4500 Q TRAP, www.appliedbiosystems.com.cn/). Following conditions were adopted for analytical measurements: UPLC: column, Agilent SB-C18 (1.8 µm, 2.1 mm*100 mm); solvent A represented the mobile phase (0.1% formic acid in pure water, and solvent B represented 0.1% formic acid in acetonitrile. A gradient program was used for sample measurement (starting with 95% A and 5% B). After 9 min, a linear gradient was programmed (5% A, and 95% B). The mixture of 5% A and 95% B was maintained (1 min). Afterward, the mixture of both A and B was attuned (95% A and 5.0% B) within 1.10 min and kept for 2.9 min. other parameters were the flow velocity (0.35 ml / min), 40 °C temperature of column oven, and 4 μl of injection volume. The effluent was alternatively connected to an ESI-triple quadrupole-linear ion trap (QTRAP)-MS.

##### ESI-Q TRAP-MS/MS

A triple quadrupole linear ion trap mass spectrometer (Q TRAP, AB4500 Q TRAP UPLC/MS/MS) system was used to obtain scans of LIT and triple quadrupole (QQQ). This QTRAP was furnished with an ESI Turbo Ion Spray interface. There were two operating modes (negative and positive ion mode). All these analyses were performed using AB Sciex software Analyst 1.6.3. The following parameters were used for ESI source operation: turbo spray ion source; ion spray voltage (IS) 5500 V (positive ion mode)/-4500 V (negative ion mode); 550 °C source temperature; curtain gas (CUR), gas II(GSII), and ion source gas I (GSI) were set at 25.0, 60, and 50 psi, respectively; High CAD (collision-activated dissociation) was used. Polypropylene glycol solutions (100 and 10 μmol/L) were used for tuning the instrument and mass calibration during LIT and QQQ modes, respectively. The scans of QQQ were obtained in the form of MRM. During this step, the nitrogen (collision gas) was fixed to a medium scale. Additional DP and CE optimization were used to perform DP and CE for individual MRM transitions.

#### Analytical methods

A statistical function (prcomp) in R (www.r-project.org) was used to execute an Unsupervised principal component analysis (U-PCA). Before U-PCA, unit variance scaling was applied to the data. The Pearson correlation coefficients (PCC) between samples were estimated using a statistical function (cor) in R and represented as heatmaps. An R package (pheatmap) was used to represent heatmaps of PCC.

##### Differential metabolites selection

A variable importance in projection value of ≥ 1 and absolute Log_2_FC (fold change) ≥ 1 are used to identify differentially (significantly) regulated metabolites. The OPLS-DA results were used to extract the variable importance in projection values and the score and permutation plots were also generated similarly. For this purpose, an R package (MetaboAnalystR) was used. Before OPLS-DA, log (log_2_) transformation and mean centering was performed for the data. A permutation (200 permutations) test was implemented to avoid overfitting.

##### KEGG annotation and enrichment analysis

The KEGG Compound database (http://www.kegg.jp/kegg/compound/) was used to identify/annotate metabolites. The KEGG Pathway database (http://www.kegg.jp/kegg/pathway.html) was used to map the annotated metabolites. Metabolites from significantly differentially regulated pathways were then subjected to metabolite sets enrichment analysis. The hypergeometric test’s p-values were used to determine the significance of metabolite sets enrichment analysis.

### Transcriptomic analyses

#### RNA extraction, library synthesis, and sequencing

A Spin Column Plant Total RNA Purification Kit (Sangon Biotech, Shanghai, China) was used according to the manufacturer’s instructions for extracting high-quality RNA from the CMC and DDC samples in triplicate. In order to ensure the quality of RNA, the following methods were used to detect the samples, and the library was constructed only after passing these tests. Agarose gel electrophoresis was performed to assess RNA integrity and DNA contamination, NanoPhotometer was used to verify the purity of RNA, Qubit 2.0 Fluorometer was employed to accurately measure the concentration of RNA, and Agilent 2100 bioanalyzer precisely detected the integrity of RNA. Later on, poly-T-attached magnetic beads were used to purify the mRNAs from the total RNAs. A fragmentation buffer was used for the conversion of the mRNAs into short fragments. A cDNA synthesis kit (ThermoFisher, Scientific, USA) was used to synthesize cDNAs from the short mRNA fragments. The AMPure XP beads were then used to tag the double-stranded cDNAs followed by repair, attachment of poly A-tail, ligation of sequencing adapter, fragment size selection, and PCR enrichment. Qubit 2.0 and Agilent 2100 bioanalyzer were then employed to assess the quality of the library. The effective library concentration (> 2 nM) was determined through a qPCR analysis. Finally, Illumina HiSeq platform (Illumina Inc., San Diego, CA, USA) was used for sequencing.

#### Computational analyses of RNA-Seq data

Quality control was performed on raw sequencing (before analyses). For this purpose, reads having adaptors, paired reads (N content > 10%), and low quality (> 50% Q ≤ 20) were removed. The GC content and error distribution were determined. It was followed by BLAST for comparison of unigene sequences with annotation databases like KEGG, NR, Swiss-Prot, GO, COG/KOG, and Tr EMBL [48]. Additionally, the amino acid sequences of unigenes were predicted. These sequences were compared with Pfam using HMMER software. The estimation of gene expression was done after transcripts splicing through Trinity (ref. sequence) and then using bowtie2 in RSEM for mapping the clean reads to each ref. sequence. The FPKM values (Fragments Per Kilobase of transcripts per Million fragments mapped) were obtained and demonstrated through “R”. The statistical analyses including PCA and Pearson correlation coefficient were performed using “R”. The screening of differentially expressed genes was executed using DESeq2 [49]. Hypothesis test correction on p-values was done through Benjamini–Hochberg method [50]. It was performed to determine the false discovery rate (FDR) and to identify the DEGs/DETs. Only those genes/transcripts were considered DEGs/DETs, which demonstrated a log_2_ fold change ≥ 1 and an FDR < 0.05. Later on, KOBAS2.0 was used to enrich the identified DEGs/DETs in KEGG pathways. An FDR value of < 0.05 was used for screening to decrease the rate of false positives in KEGG pathways prediction. Lastly, iTAK software was employed for the prediction TFs. The iTAK identifies TFs through HMM-HMM scan comparison using TF families from PlantTFDB and PlnTFDB [51].

### qRT-PCR analysis

Total RNA was extracted from the CMCs and DDCs using TRIzol reagent (Invitrogen) followed by treatment with RNase-free DNase I (Invitrogen). The cDNA synthesis and determination of relative gene expression levels were done as reported earlier [52]. The *Actin7* gene was used as an internal control to normalize the relative gene expression based on the method described by Schmittgen and Livak [53]. The specific primers used for these analyses are given in Table 1. The correlation between relative expressions and FPKM values was estimated using a statistical function (cor) in R.

**Table 1:** List of primers used for qRT-PCR analysis of selected genes in *R. glutinosa* CMCs and DDCs

Gene ID	Forward primer sequence	Reverse primer sequence
*Cluster-49959.17433*; E)-4-hydroxy-3-methylbut-2-enyl-diphosphate synthase	AATGACAAACCGACCA	TGCTTATCTCCACCCA
*Cluster-49959.18993*; (E)-4-hydroxy-3-methylbut-2-enyl-diphosphate synthase	TTATCGTAAGCCAAAGC	GAACTACAGAGCGTGAG
*Cluster-49959.59479*; 1-deoxy-D-xylulose-5-phosphate synthase	GGCACAGCACCCAATC	CCCACCGACGAAGAAC
*Cluster-49959.63438*; acetyl-CoA C-acetyltransferase	TGCGGGTCCCTTATTT	GATGTCGGCGATGGTG
*Cluster-49959.7177*; acetyl-CoA C-acetyltransferase	CTGTCGGACGAGGATT	AAGACGGGTGGAGAAA
*Cluster-59981.0*; diphosphomevalonate decarboxylase	CACCCAATCATCCAAC	TATCTCCGAAACCAGG
*Cluster-49959.1210*; cytochrome P450 family 76 subfamily A	CATCGGACAATGCTAAT	CGGAAGACGGAGGAGA
*Cluster-49959.61768*; 7-deoxyloganetin glucosyltransferase	ACGCCTTCTCGTTTAGC	TGCCACCAGGGTTTGA
*Cluster-39209.0*; 7-deoxyloganetin glucosyltransferase	ATCGTGGTCGTTGTCG	GGCTGAAGGGAGGAAA
*Cluster-61583.1*; alcohol dehydrogenase (NADP +)	TCTATGACTGGAGAACA	TGCTGACTACTGACG
*Cluster-49959.65219*; bifunctional aspartate aminotransferase and glutamate/aspartate-prephenate aminotransferase	GGTGGACAGCCAGATT	TCGGTGGACCTTATTG
*Cluster-49959.110795*; strictosidine synthase	GCGTCTGACTATTGACG	CCATCTTCTCCTTGCTGG
*Actin7*	TGGTGGAATTGATGGAA	TCATATGCATCAGGCTCG

### Co-joint analyses of metabolome and RNA-seq data

We performed a co-joint analysis between DEGs/DETs and DAMs as reported earlier [54]. The transcriptome sequencing data (DEGs/DETs expression) and metabolome profiling data (relative compound intensity of differentially regulated metabolites) were used to compute PCC, and the results were displayed as a correlation network diagram. The PCC was estimated using a statistical function (cor) in R.

### Supplementary Information

**Additional file 1: Supplementary Table 1.** List and contents of the detected metabolites in *R*. *glutinosa* DDCs and CMCs. **Supplementary Table 2.** List of differentially accumulated metabolites and their details in *R*. *glutinosa* CMCs and DDCs. **Supplementary Table 3.** Summary of the transcriptome sequencing of *R*. *glutinosa* DDCs and CMCs. **Supplementary Table 4.** Details of the pathways specific differentially expressed genes in *R*. *glutinosa* DDCs and CMCs. **Supplementary Table 5.** Differentially expressed transcription factors between *R*. *glutinosa* CMCs and DDCs.

**Additional file 2: Supplementary Figure 1.** Number of genes annotated in different databases.

**Additional file 3: Supplementary Figure 2.** Classification and enrichment of the differentially expressed genes in a) KEGG and b) GO databases.

## Data Availability

The raw data has been submitted to NCBI SRA under the project number: PRJNA871074 (https://www.ncbi.nlm.nih.gov/sra/PRJNA871074).
