# Supplementary figures and images for: Omics analyses of Rehmannia glutinosa dedifferentiated and cambial meristematic cells reveal mechanisms of catalpol and indole alkaloid biosynthesis
Source: BMC Plant Biol. 2023 Oct 5;23:463. doi: 10.1186/s12870-023-04478-3 (PMC10552359; doi:10.1186/s12870-023-04478-3)

## Slide 1
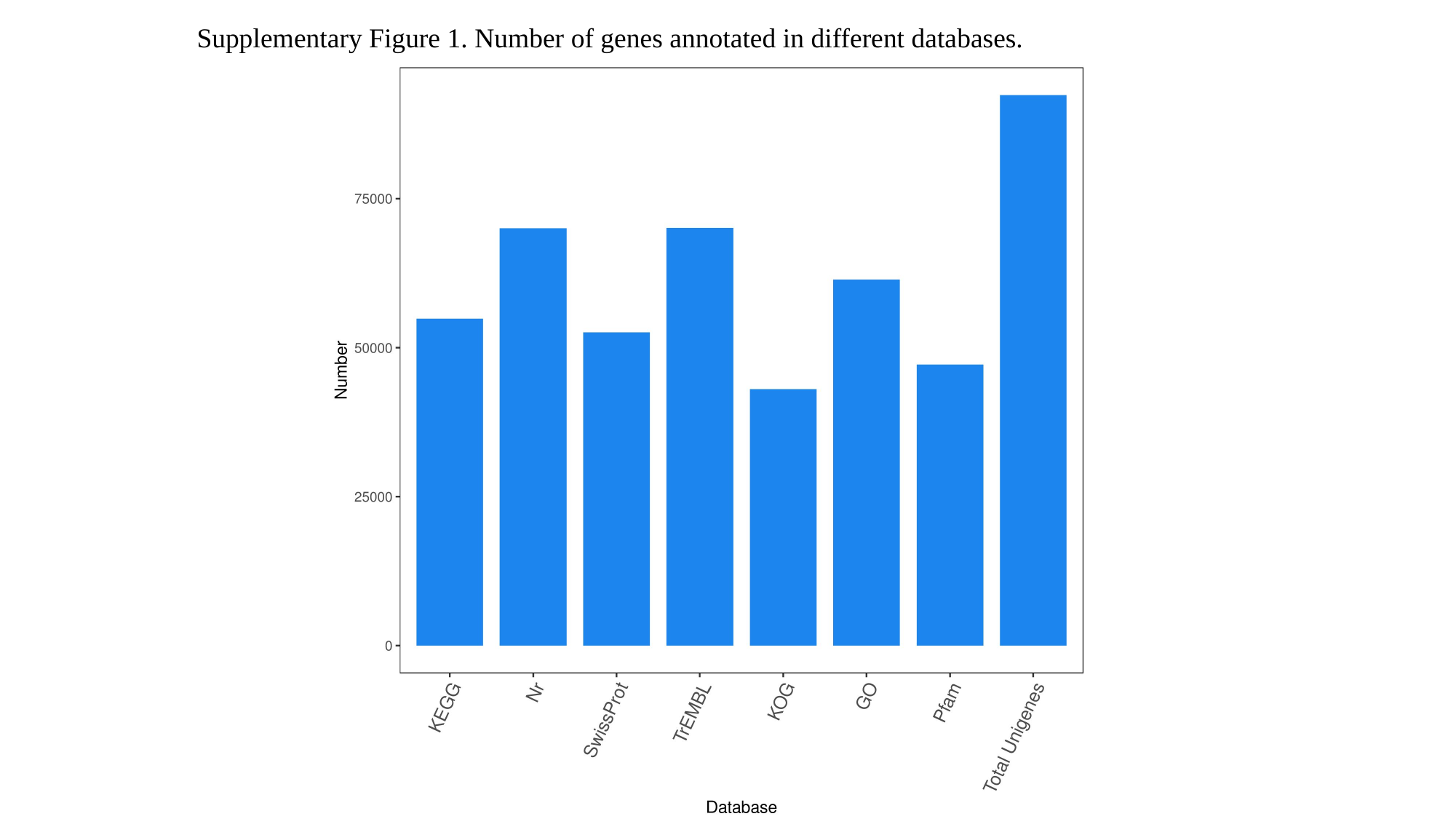

Supplementary Figure 1. Number of genes annotated in different databases.

Supplement: Supplementary file 2 — Additional file 2: Supplementary Figure 1. Number of genes annotated in different databases. [file 12870_2023_4478_MOESM2_ESM.pptx]
