# Supplementary material for: Omics analyses of Rehmannia glutinosa dedifferentiated and cambial meristematic cells reveal mechanisms of catalpol and indole alkaloid biosynthesis
Source: BMC Plant Biol. 2023 Oct 5;23:463. doi: 10.1186/s12870-023-04478-3 (PMC10552359; doi:10.1186/s12870-023-04478-3)
Supplement: Supplementary file 3 — Additional file 3: Supplementary Figure 2. Classification and enrichment of the differentially expressed genes in a) KEGG and b) GO databases. [file 12870_2023_4478_MOESM3_ESM.pptx]

## Slide 1
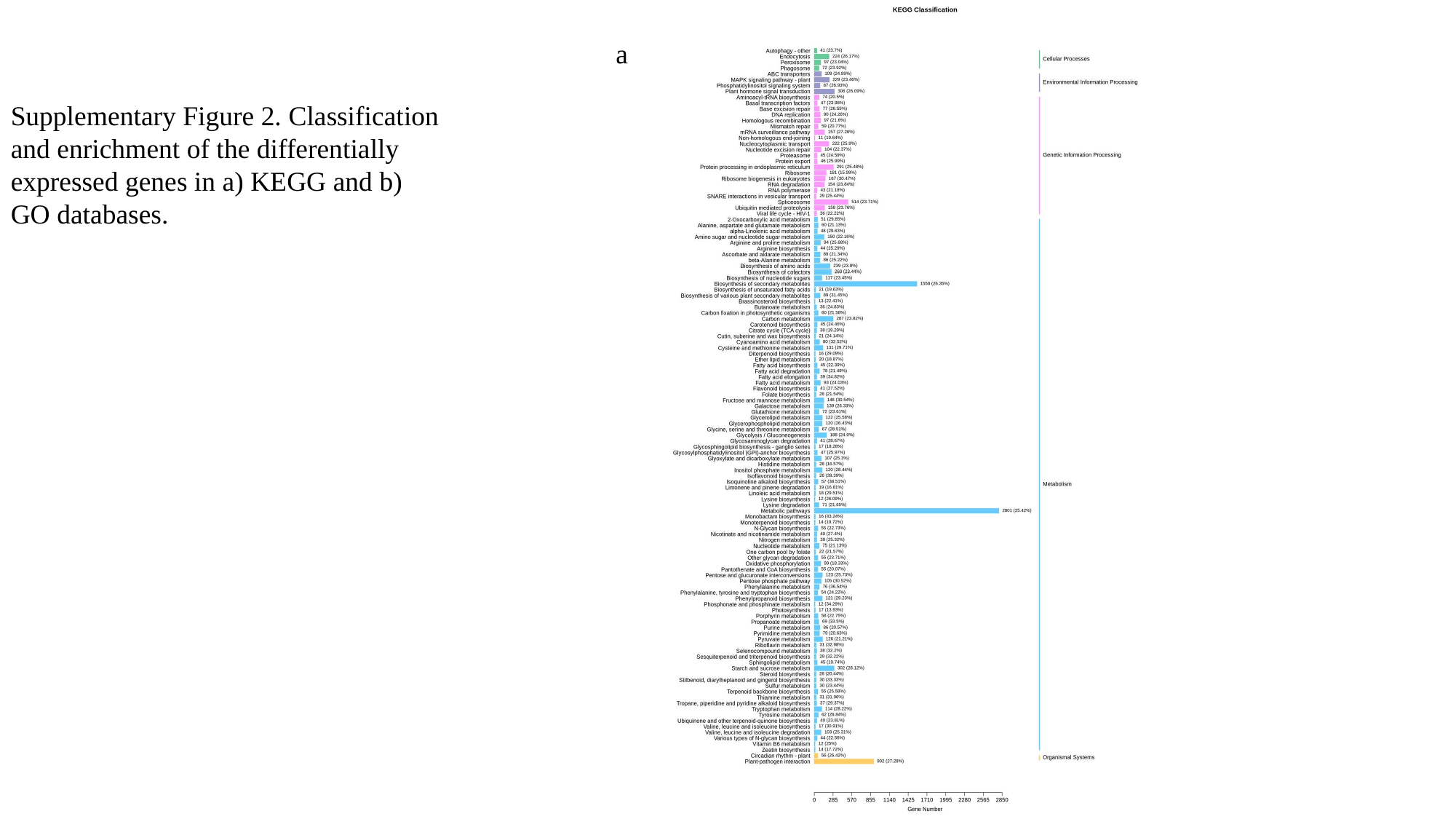

a
Supplementary Figure 2. Classification and enrichment of the differentially expressed genes in a) KEGG and b) GO databases.

## Slide 2
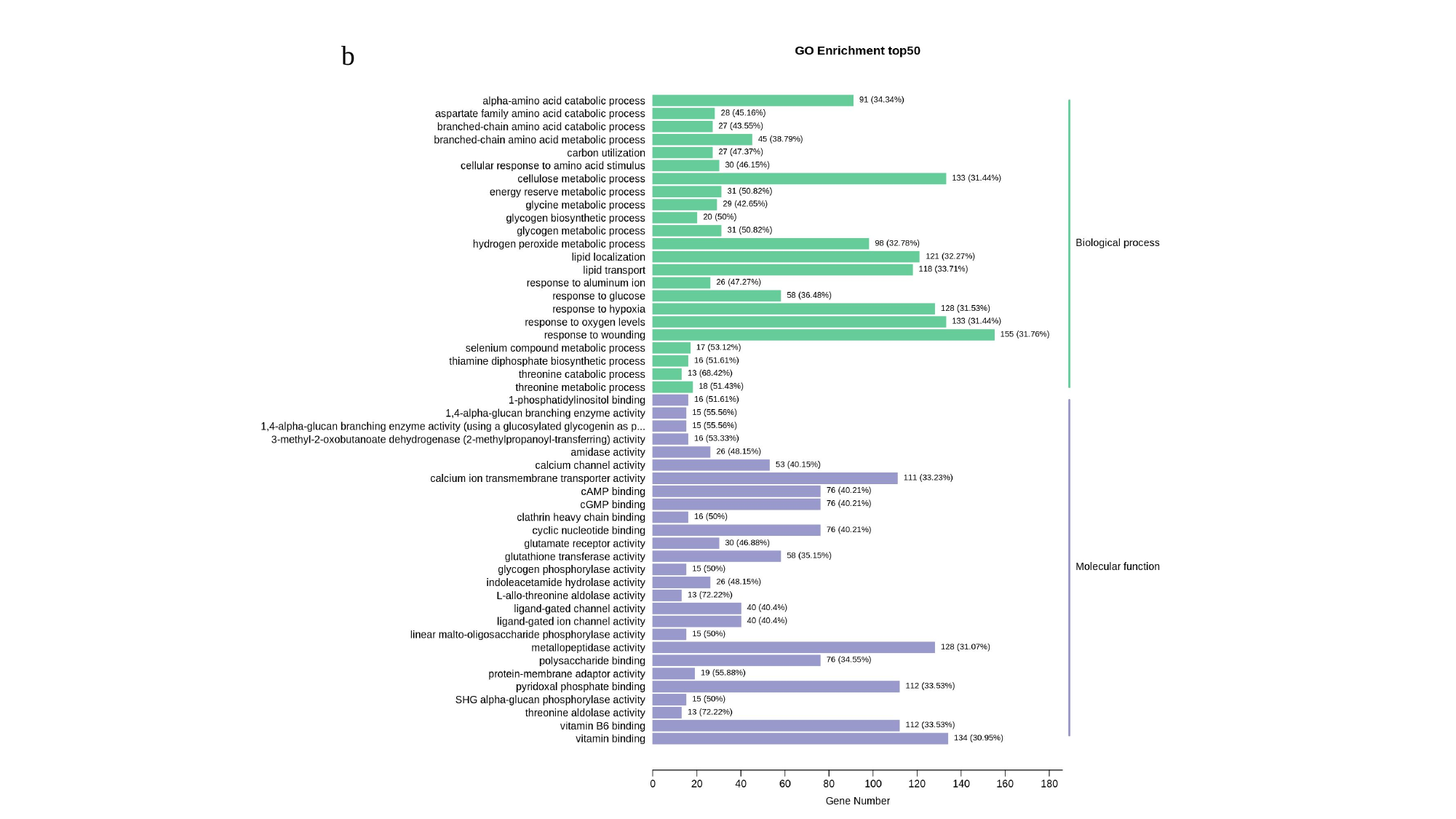

b
